# IgE-Mediated Peanut Allergy: Current and Novel Predictive Biomarkers for Clinical Phenotypes Using Multi-Omics Approaches

**DOI:** 10.3389/fimmu.2020.594350

**Published:** 2021-01-28

**Authors:** Rebecca Czolk, Julia Klueber, Martin Sørensen, Paul Wilmes, Françoise Codreanu-Morel, Per Stahl Skov, Christiane Hilger, Carsten Bindslev-Jensen, Markus Ollert, Annette Kuehn

**Affiliations:** ^1^ Department of Infection and Immunity, Luxembourg Institute of Health, Esch-sur-Alzette, Luxembourg; ^2^ Faculty of Science, Technology and Medicine, University of Luxembourg, Esch-sur-Alzette, Luxembourg; ^3^ Department of Dermatology and Allergy Center, Odense Research Center for Anaphylaxis, University of Southern Denmark, Odense, Denmark; ^4^ Department of Pediatric and Adolescent Medicine, University Hospital of North Norway, Tromsø, Norway; ^5^ Pediatric Research Group, Department of Clinical Medicine, Faculty of Health Sciences, UiT The Arctic University of Norway, Tromsø, Norway; ^6^ Luxembourg Centre for Systems Biomedicine, University of Luxembourg, Esch-sur-Alzette, Luxembourg; ^7^ Department of Allergology and Immunology, Centre Hospitalier de Luxembourg-Kanner Klinik, Luxembourg, Luxembourg; ^8^ RefLab ApS, Copenhagen, Denmark; ^9^ Institute of Immunology, National University of Copenhagen, Copenhagen, Denmark

**Keywords:** endotypes, food allergy, peanut allergy, phenotypes, predictive biomarker

## Abstract

Food allergy is a collective term for several immune-mediated responses to food. IgE-mediated food allergy is the best-known subtype. The patients present with a marked diversity of clinical profiles including symptomatic manifestations, threshold reactivity and reaction kinetics. In-vitro predictors of these clinical phenotypes are evasive and considered as knowledge gaps in food allergy diagnosis and risk management. Peanut allergy is a relevant disease model where pioneer discoveries were made in diagnosis, immunotherapy and prevention. This review provides an overview on the immune basis for phenotype variations in peanut-allergic individuals, in the light of future patient stratification along emerging omic-areas. Beyond specific IgE-signatures and basophil reactivity profiles with established correlation to clinical outcome, allergenomics, mass spectrometric resolution of peripheral allergen tracing, might be a fundamental approach to understand disease pathophysiology underlying biomarker discovery. Deep immune phenotyping is thought to reveal differential cell responses but also, gene expression and gene methylation profiles (eg, peanut severity genes) are promising areas for biomarker research. Finally, the study of microbiome-host interactions with a focus on the immune system modulation might hold the key to understand tissue-specific responses and symptoms. The immune mechanism underlying acute food-allergic events remains elusive until today. Deciphering this immunological response shall enable to identify novel biomarker for stratification of patients into reaction endotypes. The availability of powerful multi-omics technologies, together with integrated data analysis, network-based approaches and unbiased machine learning holds out the prospect of providing clinically useful biomarkers or biomarker signatures being predictive for reaction phenotypes.

## Type-I Hypersensitivity to Food

Food allergies (FA) are considered as an important public health concern (1, 2). FA can be classified into IgE-mediated, non-IgE-mediated and mixed types (3). This review focuses on IgE-mediated food allergy which is the best-known type among those food-adverse events.

### Epidemiology

There is a general perception that FA prevalence increased during the last twenty years. FA prevalence has been estimated up to 8% in the pediatric and 11% in the adult population based on a number of surveys (2, 4, 5). Beyond the sheer patient numbers, FA entails an important socioeconomic impact, causing fear of accidental exposure in patients and their families, reduced quality of life and relevant healthcare costs nearly double the amount compared to non-allergic individuals (6, 7).

### Pathophysiological Basis

IgE-mediated FA is considered as an epithelial barrier disease, resulting from food protein uptake *via* disrupted barriers (gastrointestinal tract, skin, lung) which in turn, leads to an immune dysregulation, and finally, food proteins being recognized as hostile invaders in a T helper type 2 (Th2)-skewed immune response (3, 8). During sensitization, epithelium-derived danger signals and pro-inflammatory cytokines, including interleukin 25 (IL-25) and IL-33, orchestrate the activation and expansion of type 2 innate lymphoid cells (ILC2) and dendritic cells (DCs) (9–11). Those activated DCs promote again the differentiation of naive T cells into a Th2 phenotype cells. Th2 cells and ILC2 foster the recruitment of basophils and eosinophils into the tissue beneath the epithelium (mucosa, lamina propria) through the secretion of pro-inflammatory cytokines (eg, IL-4, IL-5, IL-13) (3). Th9 cells, another effector T helper subset maturating under the influence of IL-4 and transforming growth factor beta (TGF-β), release IL-9, a cytokine which promotes the tissue accumulation of mast cells. B cell class switching to plasma cells producing food antigen-specific IgE is also fostered through IL-4 secretion by Th2 cells. Specific IgE-antibodies bind to the high-affinity IgE receptor (FcεRI) on effector cells, basophil granulocytes and mast cells (11–14). In the elicitation phase, food antigens undergo molecular interactions with cell-bound IgE-antibodies *via* specific epitopes, leading to cell activation and mediator release *via* crosslinking of FcεRI-bound IgE. Subsequently released inflammatory mediators, including histamine, prostaglandins, tryptase, and platelet-activating factor (PAF), contribute to the clinical symptoms.

### Clinical Features

Food-allergic patients present with a marked diversity by reactivity profiles (15). Clinical symptoms range from mild to severe (severity score) as does eliciting doses (sensitivity score) and time to reaction onset are highly variable (16, 17). The estimated dose likely to trigger reactions in 10% of a study population (ED_10_) vary also for specific foods (eg, peanut 11 mg; shrimp 12.8 g protein) (18). The organ involvement may relate to the skin and/or gastrointestinal tract, but also respiratory/cardiovascular symptoms in the case of potentially life-threatening anaphylaxis. Although most patients suffer from stereotypic symptoms, threshold doses depend on multiple factors under real-life conditions (atopic comorbidities; cofactors eg, exercise, alcohol, nonsteroidal anti-inflammatory drugs) (19–21). Disease prognosis and progression may also vary depending on the food allergy, such as in milk or egg allergy, which is commonly outgrown, compared to peanut allergy, which often persists lifelong (22).

### Food Allergens

A large variety of foods can cause allergic reactions and constantly, new allergenic foods are reported (23–25). The most allergenic foods include plant (peanuts, tree nuts, wheat, soy) and animal sources (milk, eggs, fish, shellfish) (13). Food allergens, the molecular drivers of allergen-specific Th2-immune responses, share molecular properties and belong to few structural protein superfamilies (26). Commonly, food allergens involved in food anaphylaxis exhibit a higher stability from digestion/processing as compared to low-allergenic homologs (27–29). Intrinsic characteristics contributing to Th2-immune modulation, such as the house dust mite allergen Der p 2 acting with auto-adjuvant properties *via* Toll-like receptor (TLR)4 signaling, are less known for food allergens (30). In-vitro models suggest that matrix effects might contribute to facilitate cross-barrier allergen uptake (eg, peanut lipids inhibiting immune-suppressive IL-10) (31). Class I food allergens are primary food allergens (eg, peanut Ara h 2). The “pollen-fruit syndrome” is mediated by specific IgE to pathogenesis-related protein-10 (PR-10; eg, birch Bet v 1) and/or profilins (eg, birch Bet v 2) as well as antibody cross-recognition of homolog class II food allergens (eg, PR-10: peanut Ara h 8). Those patients experience usually mild FA symptoms (32).

### Food Allergy Diagnosis

Usually a detailed anamnesis and IgE-tests are combined (33). In single cases, oral food challenges (OFC) are necessary, time-consuming procedures entailing a significant health risk (34). IgE (skin prick test, SPT; serum specific IgE/sIgE) is an important biomarker. Though, there is a clear trend to overdiagnosing FA as specificity of the testing is low at diagnostic cut-offs (skin wheal size diameter 3 mm, serum sIgE 0.1 kU_A_/L). Combining medical data, SPT and sIgE (extract, component-resolved sIgE) increase the diagnosis performance and might approximate OFC outcome (26, 35–37). There is the general notion that multiple IgE epitope recognition patterns correlate to FA severity and unfavorable disease progression (38, 39). Other serological parameters (e.g., total IgE, food-specific IgG4, sIgE/IgG4) are reported as discordant data. Functional assays using living cells, basophils (blood-/cell line-based) or mast cells (cell lines) feature an important added value in FA-diagnosis, although not yet being implemented into routine (29). Serum mediator levels, including histamine, tryptase and prostaglandin D2 metabolite levels, provided less consistent data like in venom- and drug-induced anaphylaxis (40). Overall, usable, reliable and affordable in-vitro predictors of clinical presentation (eg, severity, sensitivity) and risk stratification are evasive and considered still as important knowledge gaps (41). Such predictors may vary depending on the eliciting food, and therefore need to be evaluated for each food allergy.

## Emerging Omic-Areas for Clinical Endotyping

Peanut allergy (PA) is the focus of many research studies, due to its high prevalence, spectrum of clinical phenotypes, severity and lifelong duration, therefore it will be used as an example throughout. Here, we will give an overview of the immune basis for phenotype variations. We span from non-omics to omic-areas, with a focus on studies using cutting edge-technologies and studies based on patient reactivity stratification. A complete overview on biomarker approaches in peanut allergy can be found in **Table 1**.

**Table 1:** Summary of the main approaches (non-/omics) toward phenotypic biomarkers in peanut allergy based on molecular, immunological and commensal endotyping.

Method	Biomarker research area	Interpretation in peanut allergy	C E*	References**
**Molecular endotyping**				
***Non-omics***				
Immunoassays	Serum IgE to Ara h 2, Ara h 6 (less Ara h 1 and Ara h 3)	Primary peanut allergy; often, presence of sIgE and high titers (cut-off titers unequivocal) along with severity	#	(37, 38, 42–47)
	Serum IgE to Ara h 10, Ara h 11, Ara h 14, Ara h 15	Primary peanut allergy; often, presence of sIgE related to severity	#	(48)
	Serum IgE to Ara h 8	Primary sensitization to pollen (eg, birch, alder); mostly, mild reactions to peanut	#	(49–52)
	Serum IgE to Ara h 9	Primary sensitization to nsLTP (eg, peach Pru p 3); severe reactions to peanut in Ara h 1-7 negative patients	#	(53–55)
	Serum IgE to peanut epitopes	High diversity of sequential IgE-epitopes (Ara h 1, Ara h 2, Ara h 3) along with more severity	#	(38, 56–59)
	Serum peanut peptides upon in-vivo ingestion	Digestion-stable Ara h 6-peptides as candidate markers for in-vivo reactivity and serological proteomics		(60–63)
	Serum IgE-bound soluble FcϵRI	Soluble FcϵRI levels together with correlating IgE-titer as putative markers for in-vivo reactivity and severity		(64)
Integrated algorithms	Serum IgE to Ara h 2 and peanut extract, BAT together with clinical variables (eg, skin test, asthma)	Prediction of risk to experience severe events (symptoms scoring, threshold reactivity)	#	(65, 66)
***Allergenomics***				
Proteomics	Simulated digest-derived peanut peptides	Digestion-stable Ara h 2/Ara h 6-peptides as candidate markers for in-vivo reactivity and serological assays		(38, 67)
	Serum metabolomic signatures	Metabolites (eg, from dysreguated tryptophan metabolism) as candidate markers for phenotypic severity		(68)
**Immunological endotyping**				
***Non-omics***				
Immunoassays	PBMC peanut-stimulated CD4^+^ T cells	Increased Th2 cytokine expression (IL-4, IL-5, IL-9, IL-13) correlating with elevated peanut-specific IgE-titers and low threshold reactivity	#	(69) ((70))
Mass cytometry	Blood peanut-stimulated CD45^+^ cells, basophils (CD63, FceRI, CD23)	Basophil-platelet complexes (CD61, CD141, CD42b) with potential to contributing to severity and PAF-related anaphylaxis		(71) ((72, 73))
	PBMC un-/peanut-stimulated CD45^+^ cells, 11 cell types within CD4 T-cells, CD8 T-cells, B-cells, myeloid cells	Increased prevalence of activated B cells (CD19^hi^HLDR^hi^) and peanut-specific CD4 T cells (CD40L^+^CD69^+^, memory CD45RA^-^CCR7^+/-^) correlating with in-vivo reactivity		(74)
Flow cytometry	Blood peanut-/anti-IgE–induced CD63^high^ basophils (%)	Reduced basophil response and FcϵR-expression, together with low sIgE, as putative markers for severity		(52) ((75–77) (78, 79))
	PBMC peanut-stimulated CD154^+^ T cells	Increased cytokine-positive CD4^+^ T cell counts (CD154^+^CD4^+^IL-4^+^ or IL-13^+^) correlating with increased sIgE-titers and clinical threshold reactivity	#	(69, 70, 80)
		Increase of CD4^+^ T cell homing populations (CCR4: skin, lung; CCR6: mucosa; CXCR5: B cell follicle) correlating with clinical reactivity	#	(69, 81), ((82, 83))
		Increase of IL-2-dependent CD154^+^ Treg cells with regulatory (CD3^+^CD4^+^CD25^h^iCD127^low^FoxP3^+^) and memory markers (CD45RO) correlating with clinical threshold reactivity	#	(69)
		Increased coefficient of Teff/Treg (CD25^+^CD127^+/-^) correlating with clinical threshold reactivity	#	(70) ((83))
	PBMC peanut/peptide-stimulated T cells	Phenotype Th2 shift (expression gut-homing factor Integrin β7¯, CRTh2) relating to clinical reactivity		(84)
***Multimodal omics***				
Genomics	PBMC-derived peanut-activated CD154^+^ Teff/Treg (CD25^+^CD127^+/-^)	Increased diversity of the peanut-specific TCRβ repertoire (CDR3 sequences) and enrichment in the Teff compartment, in correlation with low threshold reactivity	#	(70)
	Salivary DNA-based HLA gene SNPs	With the HLA-DQB1 region confirmed as a risk factor for clinical allergy, increased odds ratios for SNPs in HLA (rs17612852, rs9275596, and rs1612904) correlate with low reaction severity	#	(85) ((86) (87, 88))
Transcriptomics	PBMC-derived peanut-activated CD154^+^ T cells	Differential gene expression patterns according to clinical phenotypes stratified by threshold reactivity, pronounced gene expression associated to Th2 and Th17 cells in individuals with low threshold reactivity	#	(69) ((70))
	Whole blood cells during food challenge	Leucocyte compositional changes (naive B cells/CD4^+^ T cells¯, neutrophils) associated with severity, upregulated “peanut severity genes” (eg, neutrophil-related function, leucocyte function) correlating with severity scores	#	(89) ((90))
Epigenetics	Whole blood CD4^+^ lymphocytes	“Peanut severity CpG” methylation associated with “peanut severity genes” (eg, immune response, chemotaxis, macroautophagy regulation; moderator genes NFKBIA and ARG1) and clinical reactivity scores	#	(90)
***Commensal endotyping***				
***Non-omics***				
Flow cytometry	Fecal microbiome	Increased IgE-binding to fecal microbes suggesting an anti-commensal Th2 response contributing to the clinical reactivity and phenotype outcome		(91)
***Microbiomics and gut issue typing***				
Genomics	Fecal microbiome	Decreased microbial richness associated with PA		(92)
		Increased alpha-diversity in low responsive individuals		(93)
		*Bacteroidales*, especially *Bacteroides fragili* increased in PA, *Clostridium* sp increased in low threshold; high *Oscillosiraceae* sp, *Lachnospiraceae* sp, *Ruminococcaceae* sp, *Frimicutes* sp and *Bacteroides* sp correlating with low threshold reactivity		(92, 93)
		*Clostridiales* abundance decreased in PA, potentially leading to a decrease in protective ROR-γt+ iTreg cell populations		(91, 92)
Transcriptomics	Gut tissue-derived peanut-specific IgE+ plasma cells	Local class switch resulting in IgE+ B cell counts correlating with serum sIgE-titers; at the gut microbiome interface, local IgE-reservoir for mast cell FcϵRI-coating and thus, candidate factors triggering clinical reactivity profiles		(94)

BAT, basophil activation test; CDR3, complementarity-determining region 3; FceRI, high-affinity IgE receptor; HLA, human leucocyte antigen; nsLTP, non-specific lipid transfer protein; PBMC, peripheral blood mononuclear cell; sIgE, specific IgE; SNP, single nucleotide polymorphism; TCR, T cell receptor; Teff, effector T cell; Treg, regulatory T cell.

*Clinical evaluation (CE), as defined here by a patient-based study design with participant severity stratification (eg, symptoms, threshold dose); #, results reported in severity stratification approach. **References in double-brackets, additional background literature based on different study design but supporting the relevance of the respective biomarker/biomarker approach.

### Molecular Endotyping

The deep analysis of allergens by proteomic technologies (*allergenomics*), based on mass spectrometry (MS), pushed the boarders of knowledge around allergenic peanut proteins including basic aspects of primary structures and post-translational modifications (95–97).

#### Non-Omics

**Figure 1:**
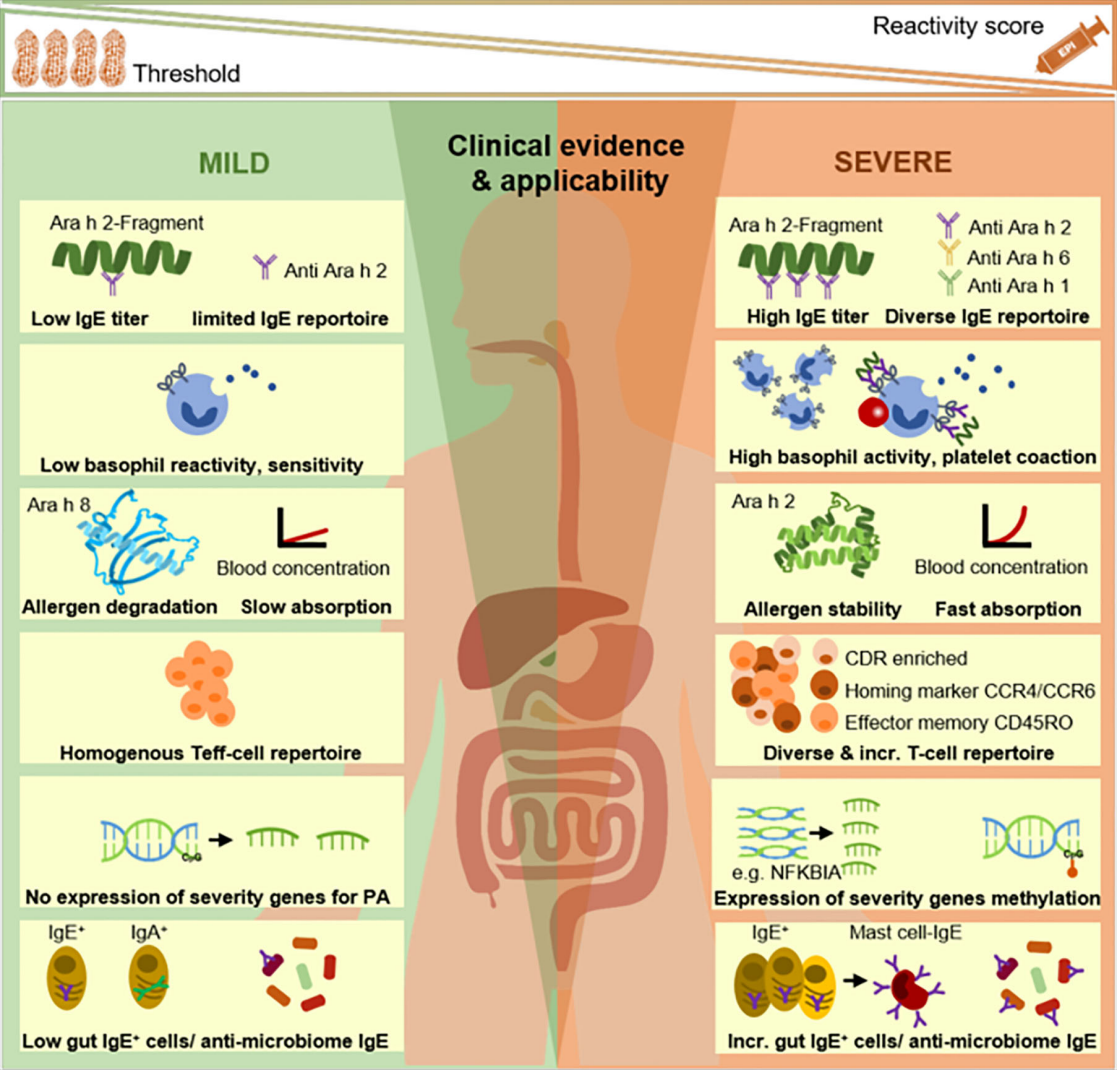
Endotyping of peanut-allergic patients: from selected, established to newly discovered approaches. The association of IgE-signatures (IgG-profiles not shown/reviewed in text) and basophil reactivity profiles with clinical phenotypes is widely established. Research fields on peripheral allergen tracing, deep immune typing (eg, T cells), gene expression/modification as well as local gut immune responses and gut microbiome-host interactions represent putative endotyping axes which require further investigations and finally, systems-level integration in future studies. Incr., increased; PA, peanut allergy.

A total of 18 unique peanut iso/allergen are reported (with Ara h 4 now being considered an isoallergen of Ara h 3) (98). Serum IgE-reactivity to seed storage proteins, including 2S albumins (Ara h 2, Ara h 6) and cupins (Ara h 1, Ara h 3) relates to primary PA. Cross-reactivity markers are the PR-10 protein Ara h 8 (birch allergy) and the non-specific lipid transfer proteins (nsLTP) Ara h 9 (peach-related fruit allergy). The diagnostic relevance of molecular vs extract-based IgE-signatures seems to vary for patient groups from different geographic origins (42, 99–101). However, a recent meta-analysis summarized the overall high diagnostic accuracy of sIgE to Ara h 2 in terms of sensitivity and specificity (95% CI 75.6, 88.9 and 95% CI 77.4, 88.4, respectively) at a cut-off of 0.35 kU_A_/L (37). Patients are often IgE-positive for both Ara h 2 and Ara h 6. Recently, Ara h 2 was described as the immunodominant molecule among the two allergens, with higher capacity to activate in-vitro effector cells (basophils, mast cells), pointing to a greater role of Ara h 2 in both disease pathophysiology and as diagnostic severity marker (**Figure 1**) (43). Even allergen peptides can be beneficial. Indeed, systematic peptide-based scanning approaches (epitope mapping) revealed that increasing IgE-epitope diversity correlated with a more severe phenotype (38, 67). In-vitro basophil activation tests (BAT), using basophils from peanut-allergic patients challenged with peanut protein, revealed dose-dependent activation (%-CD63+ basophils). High performance to identify clinical PA (98.7% specificity, 74.7% sensitivity) and high precision to identify individuals with severe outcome (97% specificity, 100% sensitivity) has been recently reported for large UK study populations (**Table 1**) (65). Here, the best prediction of low threshold reactivity was determined in a multivariate statistical tool combining various parameters, SPT, sIgE (Ara h 2, peanut extract), peanut extract-sIgG4/IgE quotient and BAT. In a similar integrated approach, a predictive algorithm based on the CD63 ratio (BAT with peanut protein) and clinical parameters (eg, exercise-induced asthma), had been proposed to predict severe reactions (66).

#### Allergenomics

As an extension to allergenomics as an analysis of the allergen repertoire of an allergen source, a new research axis applied proteomic approaches to study in-vitro degradation patterns of peanut digests by simulating gastric or small intestine milieus (102, 103). The pronounced digestion stability of Ara h 2, 6, and specific peptides was linked to IgE-recognition and suggested as triggers of the immune response in-vivo. Those peptide structures might be novel candidates for serological assays, be it as antigens in immunoassays or as references for peptide identification in patient blood. In fact, upon ingestion, peanut allergens are degraded, followed by absorption across biological barriers and distribution *via* the bloodstream (20, 60, 104–106). The analysis of allergen residues in human samples after peanut ingestion has been recognized as an important challenge (60, 61). Recent antibody-based studies, combined with removal of interfering endogenous immunoglobulins, succeeded to detect peanut allergens in a reliable fashion (62). Proteomic analyses of such peripheral allergen peptides, together with MS-based analyses of serological metabolomics signatures (68), might be promising avenues toward molecular endotyping of peanut-allergic patients, and toward marker discovery for phenotype prediction (**Figure 1**).

### Immunological Endotyping

#### Non-Omics

Deep immunological endotyping, including aspects of the genome, epigenome, transcriptome and proteome provided insights in the immune landscape of PA phenotypes, with prospect of future multimodal omics, meaning the integration of heterogeneous data from those sources.

Tordesillas et al. studied the in-vitro activation of granulocytes (basophils, eosinophils, neutrophils), monocytes, dendritic cells, T cells, B cells and NK cells in whole blood using single-cell mass cytometry (71). More B cells and eosinophils but less neutrophils were found in resting CD45^+^ cells from peanut-allergic vs healthy individuals. After stimulation with peanut protein, the highest response was observed for basophils (CD16, CD23, CD63), but also monocytes, dendritic cells and neutrophils became activated, pointing to an emerging role of these myeloid cells related to clinical PA. Elevated plasma PAF levels had been associated with increased severity of PA earlier (72, 73). Basophils were found to form physical complexes with platelets (CD61, CD141, CD42b) upon peanut activation, suggesting a novel way of PAF-related anaphylaxis (**Figure 1**) (71). Neeland et al. applied mass cytometry to study peripheral immune signatures associated with clinical PA, using peripheral blood mononuclear cells (PBMC) from peanut-allergic and controls (74). Allergic infants distinguished from sensitized infants by increased prevalence of a B cell cluster (CD19^hi^HLDR^hi^). Upon in-vitro stimulation with peanut protein, increased levels of CD4 T cells (CD40L^+^CD69^+^, memory CD45RA^-^CCR7^+/-^) discriminated peanut-allergic from controls.

Other studies focused on analyzing the T cell compartment in PA (81, 107–109). Chiang et al. compared peanut-allergic (OFC positive at <1g cumulative peanut dose), high-threshold (OFC negative at ≤1g cumulative peanut dose but sIgE to peanut and clinical PA history), and healthy individuals (69). In-vitro PBMC stimulation induced a significant increase in peanut-responsive T cells (CD154^+^CD4^+^) and significant cytokine increases (mainly IL-4, IL-13) in peanut-allergic patients only. Cytokine-positive T cell counts (CD154^+^CD4^+^IL-4^+^ or IL-13^+^) correlated with sIgE-titers. Peanut-allergic patients had higher shares of peanut-activated Th2 cells with homing markers (CCR4: skin, lung; CCR6: mucosa; CXCR5: B cell follicle) as compared to controls. Peanut-responsive T cells presented with surface marker heterogeneity as well as enrichment for effector memory T cells (CD45RO) and regulatory marker expression (CD3^+^CD4^+^CD25^hi^CD127^low^FoxP3^+^, delayed IL-2-dependent activation). RNA sequencing of peanut-activated T cells confirmed proinflammatory Th2-polarization with multicytokine expression. This study pointed to the heterogeneous nature of the peanut-specific Th2 response in presence of functional Treg cells. The lack of T cell reactivity (peanut-specific Th2, Treg) in high-threshold individuals discriminated those from peanut-allergic individuals. In a similar approach, Ruiter et al. investigated the T cell response to peanut protein comparing peanut-allergic (OFC positive at <0.5g cumulative peanut dose) and hyporeactive (OFC negative at ≤0.5g cumulative peanut dose but clinical PA history) individuals (70). Compared to hyporeactive patients, stimulated PBMC from peanut-allergic patients showed a higher CD154^+^CD4^+^ T cells response and stimulation index correlating with elevated peanut-specific CD4^+^ T cell and complementarity determining region 3 (CRD3; T cell receptor domain identified by RNA sequencing) counts. Indeed, CDR3 constitutes the most critical region responsible for recognizing processed antigens (110, 111). Some peanut-specific CRD3 (17%) were found exclusively in CD154^+^CD4^+^ T cells from peanut-allergic individuals (70). CRD3 were also more variable in effector T cells (CD25^+^CD127^+^) than Treg cells (CD25^++^CD127^-^), suggesting skewing toward a compartment with expanded effector T cell repertoire in allergic but not in hyporeactive patients. The ratio of peanut-specific effector T cell vs Treg discriminated individuals stratified by threshold doses. The pronounced clinical reactivity of peanut-allergic patients was concluded to correlate to peanut-specific effector T cells characteristics (frequency, proportion, reactivity), rather than a defective Treg response (**Figure 1**).

#### Multimodal Omics

Gene sequencing studies provided insights into FA immune regulation and epithelial barrier function (112, 113). Genome-wide association studies reported on loci correlated to specific FAs (**Table 1**) (85–87, 114). Beyond aspects of disease susceptibility, recent studies focused on genes involved in acute inflammation in PA patients. Watson et al. analyzed the time-resolved transcriptome in peripheral blood sampled from peanut-allergic individuals during OFC (at baseline, 2 h/4 h later), comparing peanut vs placebo (89). Indeed, specific gene expression changes were induced by peanut intake. Gene upregulation was commonly found (1,411/2,168 genes) correlating with evolving peanut-induced inflammation. In leucocyte cell subsets after deconvolution, resting macrophages (M0) and neutrophilic granulocytes increased while naive CD4^+^ T cells decreased during OFC. Genes associated with peanut-allergic reactions were mostly found in a co-expression module with upregulated genes related to inflammatory processes. Six key driver genes were identified (3/6 with established role in inflammation) as modulators of the peanut-reactive co-expression module. The data-driven approach on genes involved in peanut-allergic reactions was further developed by Do et al. using transcriptome analysis during OFC (at baseline, 2 h/4 h later), in combination with baseline epigenomic profiling (90). The participants’ clinical reactivity was stratified by threshold-weighted severity grades. More than 300 genes (“peanut severity genes”) had significant expression changes during OFC and were found to be associated with reaction severity. Biological processes related to upregulated peanut severity genes clustered by function, mostly around neutrophils (activation, degranulation, neutrophil-mediated immunity). With pronounced reaction severity, neutrophilic granulocytes increased also in number while naive CD4^+^ T cells and naive B cells decreased significantly during the course of OFC. Most peanut severity genes clustered together by co-expression. Gene interaction network analysis indicated the central role of two genes, NFKBIA (NF-kappa-B-inhibitor alpha, a regulator protein) and ARG1 (arginase, a catabolic enzyme and immune regulator), on reaction severity. Epigenetic modification correlating with reaction severity, as measured by methylation signatures of CpG dinucleotides in CD4^+^ lymphocytes, was found for more than 200 CpGs (“peanut severity CpG”). A causal relationship between methylation and peanut severity genes gene expression was established, pointing further to the relevance of epigenetic modifications in the context reaction severity.

### Commensal Endotyping

The gut represents the largest interface for the interaction between the human body and food allergens. There is a constant regulatory interaction between the immune system and the intestinal microbiome (115). The microbiome might promote Th2-immunity to food by regulating eosinophils (frequency, function) in the gut (116).

#### Non-Omics

More recently, sIgE-binding to commensal bacteria was discovered in food-allergic children, suggesting structural similarities between food allergens and microbial structures (91). Molecular mimicry of the microbiome plays a role in a number of inflammatory diseases, such as celiac disease. Here, structures on P*. fluorescens*, a commensal which is overrepresented in celiac disease, have been found to mimic human leukocyte antigen (HLA) locus HLA-DQ2.5- and activate mucosal T cells, suggesting a pathological dysfunction of the gut barrier (117). First studies comparing amino-acid sequence similarities between known food allergens and microbiome data revealed conserved regions of T-cell immune recognition on commensal bacteria (118). Carrasco Pro et al. further showed similarities between human microbiome sequences and inhalation allergens (119).

#### Microbiomics and Gut Tissue Typing

The adaptive immune response is influenced by microbial interactions with secreted IgA (120), together with lower richness and lower local species diversity (alpha diversity), accompanied by a dysbiosis of commensal strains (92, 93). A previous study demonstrated that peanut allergy is marked by higher Bacteroidales, especially *Bacteroides fragilis* and reduced Clostridiales abundance (92). Low threshold reactivity to peanut has been connected to an increase in *Clostridium* sp, *Oscillosiraceae* sp, *Lachnospiraceae* sp, *Ruminococcaceae* sp, *Frimicutes* sp, and *Bacteroides* sp (91–93).

At which gastrointestinal sites immune dysregulation and allergic sensitization might develop is unexplored. Recently, large numbers of allergen-specific B cells were described in the gut (stomach, duodenum) of peanut-allergic patients (94). These IgE^+^ cells are rarely found in the blood (121, 122). Gut IgE^+^B cells counts were found to correlate with serum IgE-titer concentrations. Importantly, inter-individual variations in this local IgE^+^B but also variable mast cell IgE-loading by different IgE^+^B clones might explain differential reaction phenotypes in peanut-allergic patients’ reservoir (**Figure 1**). The high number of IgE^+^ B cells in the gut combined with increased intestinal permeability might explain the high sIgE-levels found in fecal samples of food-allergic patients (123).

These findings give an idea of a new mechanism in which the microbiome may initiate, trigger and influence allergic reactions. This in turn may lead to novel ways to stratify patients, due to their metaproteomic profile, as has been shown for other inflammatory diseases (124, 125).

## Conclusion: Perspective Toward New Integrative Approaches

Deciphering the immunological response to food proteins shall enable the stratification of patients into reaction endotypes, for advanced understanding of their phenotypic heterogeneity. The ambitious but ultimate goal will be to identify clinically useful predictors for allergic reactions to food, with emphasis on predicting clinical outcome, severity and threshold dose, upon allergen exposure in order to adapt avoidance protocols and symptomatic medication (15, 126). Recent PA-studies demonstrated the complexity of the immune mechanism, as investigated during simulated allergen-specific stimulations or during the course of clinical reactions (71, 74, 89, 90). Several studies did even compare immune targets in individuals with variable clinical reactions, based on severity or sensitivity (69, 70, 90). To explain clinical manifestations of reaction phenotypes, various aspects are considered of fundamental relevance, including the molecular IgE-signature/-repertoire, the potency/repertoire of effector cells, the kinetics of allergen degradation/absorption, allergen-specific T cell reactivity profiles, genes/methylations and aspects of the gut microbiome including composition and host interaction (**Figure 1**). The availability of multiple omics technologies, proteomics, high-dimensional mass cytometry, transcriptomics and epigenomics, allowed identifying promising molecular and immunological targets for future human studies. Taken individually, each omics-approach has assets and drawbacks (reviewed by (127, 128) but together they might unfold their full potential. Unbiased machine-learning, integrated data analysis of heterogeneous datasets as well as network-based approaches will be required to establish algorithms for providing insights in disease pathophysiology and for inferring biomarkers or biomarker signatures being predictive for reaction phenotypes (33, 127, 129, 130). Finally, those insights shall advance the stratification of individuals prior to selection for oral immunotherapy or early food introduction for prevention, both pioneer areas research in PA (131, 132).

## Author Contributions

RC and JK wrote the manuscript. MS, FC-M, and CB-J revised the manuscript as to clinical content. CH, PW, PS, and MO provided critical feedback to the manuscript concept and scientific content. AK developed the review concept together with RC/JK and helped to shape the manuscript. All authors contributed to the article and approved the submitted version.

## Funding

Supported by the Luxembourg National Research Fund on PRIDE program grants PRIDE/11012546/NEXTIMMUNE and PRIDE17/11823097/MICROH; supported by the Personalized Medicine Consortium grant APSIS, PMC/2017/02 and by the Ministry of Research, Luxembourg.

## Conflict of Interest

PS declares being a scientific advisor in RefLab ApS. CB-J declares being a Clinical Investigator for Novartis, Aimmune, Hal Allergy, Allakos, and Miltenyi.

The remaining authors declare that the research was conducted in the absence of any commercial or financial relationships that could be construed as a potential conflict of interest.
